# Identification of co-expression hub genes for ferroptosis in kidney renal clear cell carcinoma based on weighted gene co-expression network analysis and The Cancer Genome Atlas clinical data

**DOI:** 10.1038/s41598-022-08950-2

**Published:** 2022-03-21

**Authors:** Shengxian Li, Ximei Xu, Ruirui Zhang, Yong Huang

**Affiliations:** 1 National Center for International Research of Bio-Targeting Theranostics, Guangxi Key Laboratory of Bio-Targeting Theranostics, Collaborative Innovation Center for Targeting Tumor Diagnosis and Therapy, Guangxi Talent Highland of Bio-Targeting Theranostics, Guangxi Medical University, Nanning, 530021 Guangxi China; 2 National Center for International Research of Biological Targeting Diagnosis and Therapy, Guangxi Key Laboratory of Biological Targeting Diagnosis and Therapy Research, Collaborative Innovation Center for Targeting Tumor Diagnosis and Therapy, Guangxi Medical University, Nanning, 530021 Guangxi China

**Keywords:** Cancer, Computational biology and bioinformatics

## Abstract

Renal clear cell carcinoma (KIRC) is one of the most common tumors worldwide and has a high mortality rate. Ferroptosis is a major mechanism of tumor occurrence and development, as well as important for prognosis and treatment of KIRC. Here, we conducted bioinformatics analysis to identify KIRC hub genes that target ferroptosis. By Weighted gene co-expression network analysis (WGCNA), 11 co-expression-related genes were screened out. According to Kaplan Meier's survival analysis of the data from the gene expression profile interactive analysis database, it was identified that the expression levels of two genes, PROM2 and PLIN2, are respectively related to prognosis. In conclusion, our findings indicate that PROM2 and PLIN2 may be effective new targets for the treatment and prognosis of KIRC.

## Introduction

Renal cell carcinoma (RCC) is known to account for more than 90% of all adult kidney tumors^1^. Its predominant histological subtype is renal clear cell carcinoma (also called kidney renal clear cell carcinoma; KIRC), which accounts for ~ 80% of RCC^2^, the prognostic risk assessment of KIRC varies according to TNM staging, age, and gender. The five-year survival of patients is closely related to the pathological grade, depending on whether the tumor is early, intermediate, or advanced. If the patient is in the early stage of the disease, i.e., a pathological grade of I or II, the patient will have a better treatment effect and a longer survival period after surgical resection, with a five-year survival rate as high as 80–90%. If the patient is in the middle and advanced stages of KIRC, with a pathological grade of III or IV, the prognosis of the patient is relatively poor, tumor malignancy and the probability of metastasis and recurrence high, and the survival period shortened to five years. In these instances, the survival rate is ~ 20%^3–5^. Therefore, it is recommended that patients must be detected and treated early. However, only ~ 10% of patients with KIRC show characteristic clinical symptoms, and 20–30% of patients have metastases already detected at the first visit. In ~ 30% of patients with localized KIRC, recurrence occurs twice after surgical resection, whereas conventional radiotherapy and chemotherapy are furthermore largely ineffective in curing KIRC^6^. Although the mechanism of its occurrence and development has been studied in-depth, its pathogenesis and carcinogenesis are still unclear, and specific sensitive tumor markers are still lacking^7^. Therefore, it is imperative to improve our understanding of the molecular mechanism of KIRC to identify prognostic biomarkers and treatment targets that can guide the existing clinical phenotypic staging systems.

Modern high-throughput sequencing technologies include DNA sequencing, RNA sequencing (RNA-seq), and epigenome research. Bioinformatics analysis of the results of these technologies, especially network analysis, allows us to obtain genome assembly, genome annotation, and gene function annotation^8–10^. Bioinformatics provides a new perspective by efficient integration of multiple large-scale datasets for various human diseases. However, most bioinformatics research only focuses on identifying differentially expressed genes (DEGs)^11^, which neglects the functional relationship and high correlation between genes with similar expression patterns^12,13^. Weighted gene co-expression network analysis (WGCNA) explores the correlation between different genomes or between samples and clinical features by constructing a free-scale gene co-expression network^14–16^. This method is widely used to identify related clinical modules and hub genes of different types of cancer^17^. For example, Yuan et al. used WGCNA as early as 2017 to discover that the expression of six pivotal genes is closely related to the progression and prognosis of KIRC^18^. Lastly, Zhang et al. used WGCNA to verify the six hub genes of colorectal cancer^19^.

Ferroptosis is a cell death pathway, first proposed by Professor Brent Stockwell of Columbia University in 2012^20^, which is caused by an increase in iron ion load driving a large amount of lipid peroxidation. It primarily involves three major metabolic mechanisms, including iron metabolism, lipid metabolism, and amino acid metabolism^21^. Among these, lipid metabolism induces tumor development and treatment resistance by enhancing lipid synthesis, storage, and catabolism. In general, the membrane fatty acid composition, such as the ratio of saturated fatty acids, monounsaturated fatty acids, and polyunsaturated fatty acids, promotes cell growth. However, tumor cells show plasticity in fatty acid metabolism which has resulted in a shift in research focus to limit fat toxicity and ferroptosis as a means to improve overall survival (OS) in patients with cancer^22,23^.

Studies have shown that ferroptosis is closely related to the occurrence and development, as well as prognosis and treatment of tumors—with KIRC as a tumor type sensitive to ferroptosis^24^. In KIRC and normal tissues, ferroptosis regulators are related to *PD-L1* expression which affects the tumor immune microenvironment promoting tumorigenesis^25^. Furthermore, Wu et al. showed that ferroptosis is related to the clinicopathological characteristics of patients with KIRC and constructed five KIRC and ferroptosis-related prognostic models^26^. Another study found that 11 ferroptosis genes (*CARS*, *CD44*, *DPP4*, *GCLC*, *HMGCR*, *HSPB1*, *NCOA4*, *SAT1*, *PHKG2*, *GOT1*, *HMOX1*) were significantly related to the OS of patients with KIRC^27^. Moreover, *CHAC1*^28^, Acyl-CoA Thioesterase 8 and 11^29^, and *SUV39H1*^30^ may be effective prognostic indicators of KIRC. Lastly, Gao et al. found that the TAZ/WNT10B axis may serve as biomarkers and therapeutic targets for KIRC immunotherapy^31^. Taken together, there are many studies on KIRC, but the current understanding of its pathogenesis, tumor progression, and metastasis is still imperfect, with many of its characteristics differing from other cancers^32–34^. Therefore, finding novel ferroptosis-related targets or pathways for the treatment of KIRC would two-fold curb the high recurrence rate and growing drug resistance of KIRC.

In this study, we downloaded the RNA-seq data of KIRC samples from The Cancer Genome Atlas (TCGA), used WGCNA to determine the genes and modules related to the clinical characteristics of patients with KIRC, and aimed to identify the co-expression of genes related to clinical characteristics and ferroptosis. Then, through survival analysis of the co-expressed genes, we determined hub genes with the highest prognostic potential. Our study aims to find a link between KIRC-related genes with ferroptosis that may be used for the prognosis of KIRC. This could be insightful for the development of potential biomarkers and treatment targets, providing a new perspective for future research into KIRC.

## Materials and methods

### Data sources and study design

**Figure 1:**
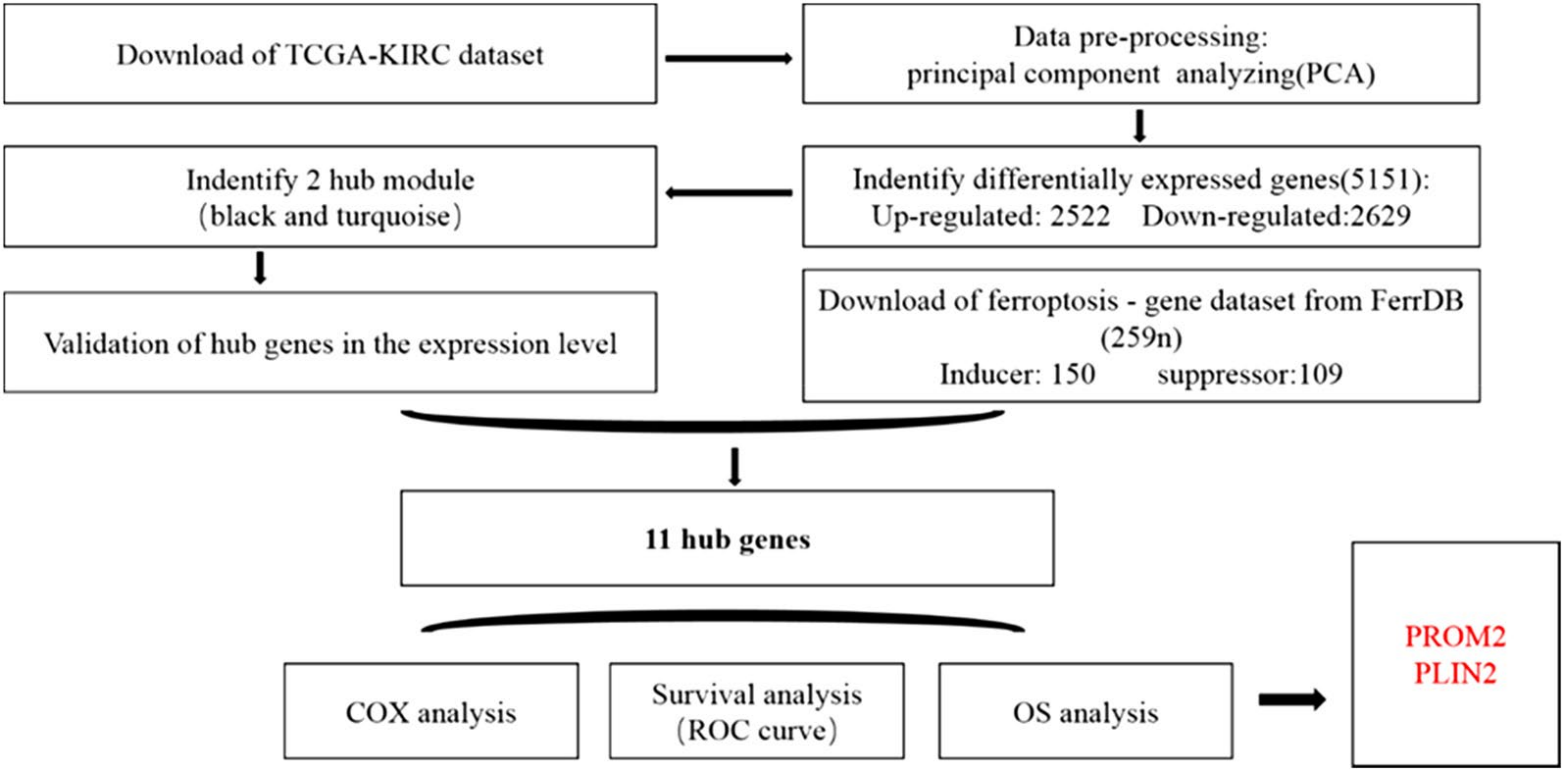
Work-flow of this study.

RNA-seq data and clinical information on patients were downloaded on October 17th, 2021 from the ‘Colon and Rectal Cancer’ cohort of the TCGA database (https://www.cancer.gov/about-nci/organization/ccg/research/structural genomics/tcga), hosted at the Xena website of the University of California at Santa Cruz^35^ (http://xena.ucsc.edu/; Table 1 and Supplementary Tables S1–S2). The RNA-seq data contained 538 tumor samples and 407 normal tissue samples from 945 patients with KIRC. Data on the clinical information, including the OS of patients with KIRC, were also obtained from the TCGA database (http://xena.ucsc.edu/; Supplementary Tables S3). In addition, data on genes related to induction and inhibition of ferroptosis were downloaded from the FerrDB database (http://www.zhounan.org/ferrdb/; Supplementary Table S4). Using principal component analysis (PCA), we excluded samples if the first two principal components identified were unable to distinguish tumor tissue from normal tissue. The study process is shown in Fig. 1.

**Table 1:** The clinical information and sample size for TCGA KIRC dataset.

Characteristics	LIVING (*N* = 605)	DECEASED (*N* = 340)	Total (*N* = 945)	FDR
**Cancer type**				
Kidney clear cell renal carcinoma	605 (64.02%)	340 (35.98%)	945 (100.00%)	
**Age**				
Mean ± SD	58.74 ± 11.72	64.53 ± 11.82	60.82 ± 12.08	
Median [min, max]	59.00 [26.00, 86.00]	64.00 [32.00, 90.00]	61.00 [26.00, 90.00]	
**Gender**				0.36
Female	203 (21.48%)	125 (13.23%)	328 (34.71%)	
Male	402 (42.54%)	215 (22.75%)	617 (65.29%)	
**Stage**				9.20E-44
Stage I	369 (39.17%)	87 (9.24%)	456 (48.41%)	
Stage II	72 (7.64%)	25 (2.65%)	97 (10.30%)	
Stage III	133 (14.12%)	98 (10.40%)	231 (24.52%)	
Stage IV	30 (3.18%)	128 (13.59%)	158 (16.77%)

### PCA analysis

The theoretical basis for realizing high-dimensional data visualization is based on dimensionality reduction algorithms. UMAP, tSNE, and PCA are several common dimensionality reduction methods used in our study.

For UMAP, we used the R software package UMAP v0.2.7.0 for analysis. The Z-score was first performed on the expression spectrum, followed by the UMAP function for dimension reduction analysis to obtain the matrix. For PCA, the R software package STATS v3.6.0 was used for analysis. After obtaining the Z-score, the prCOMP function was used for dimension reduction analysis to obtain the matrix. For tSNE, the R software package Rtsne v0.15 was used for analysis. Similarly, the Z-Score was first performed on the expression spectrum, and then the Rtsne function was used for dimension reduction analysis to obtain the matrix^36,37^. Of these three methods, UMAP proved to be better than the rest. UMAP uses a very efficient visualization and scalable dimensionality reduction algorithm. In terms of visualization quality, the UMAP algorithm has a competitive advantage over t-SNE, but also retains more global structure, has superior running performance, and better scalability. Furthermore, UMAP has no computational limit on the embedding dimension, which makes it useful as a general-purpose dimension reduction technique for machine learning. UMAP also provides useful and intuitive properties that preserve more global structure, especially the continuity of cell subsets. Therefore, we used UMAP to exclude samples by distinguishing tumors from normal tissue.

### Identification of KIRC DEGs

To obtain the DEGs for differential analysis between different groups, we used the R software package-Limma (Linear Models for Microarray Data, DOI:10.1093/NAR/GKV007, v3.40.6), which is a DEG screening method based on generalized linear models. Specifically, we obtained the expression spectrum dataset and used the ‘lmFit’ function to perform multiple linear regression. Furthermore, we used the ‘eBays’ function to compute moderated t-statistics, moderated f-statistic, and log-odds of DEGs by empirical Bayes moderation of the standard errors towards a common value to finally obtain the DEG significance of each gene. DEGs were defined as those showing |log2(fold-change)|> 1 and *P* < 0.01. Volcano plots of DEGs were plotted using the R function 'ggplot2'.

### WGCNA

At first, the Pearson's correlation matrices and average linkage method were both performed for all pair-wise genes, and then a weighted adjacency matrix was constructed using a power function *A*_*mn*_ =|*C*_*mn*_|^β^ (*C*_*mn*_ = Pearson's correlation between genes *m* and *n*; *A*_*mn*_ = adjacency between genes *m* and *n*)^17,38^; and *β* was a soft-thresholding parameter that could emphasize strong correlations between genes and penalize weak correlations. After choosing the power of 6, the adjacency was transformed into a topological overlap matrix (TOM)^39^, which could measure the network connectivity of a gene defined as the sum of its adjacency with all other genes for network generation, and the corresponding dissimilarity (1-TOM) was calculated. To classify genes with similar expression profiles into gene modules, average linkage hierarchical clustering was conducted according to the TOM-based dissimilarity measure with a minimum size (gene group) of 100 for the genes dendrogram, with the sensitivity set to 4. To further analyze the module, we calculated the dissimilarity of module eigengenes, chose a cut line for module dendrogram and merged some modules^40^. In addition, we also merged modules with a distance of less than 0.25, and finally obtained nine co-expression modules. It is worth noting that the grey module is considered to be a gene set that cannot be assigned to any module.

### Correlation analysis of clinical features and modules for identification of KIRC hub genes

Using clinical features as input, we performed correlation analysis between the modules and clinical features, as well as between MM (module membership) and GS (gene significance). Based on the weighted correlation, a hierarchical clustering analysis was performed, and the clustering results were segmented according to set criteria to obtain different gene modules—represented by the branches and different colors of the clustering tree. The relationship between models was studied and an interaction network of different models was constructed on the system level. We used the “Venn” R package to draw a Venn map of ferroptosis-related DEGs and prognostic genes, while also preserving information related to the intersection genes.

### Functional enrichment analysis by gene set enrichment analysis (GSEA)

The GSEA v3.0 software was obtained from the GSEA (http://software.broadinstitute.org/gsea/index.jsp) website^36^, whereas the c2.cp.kegg.v7.4.symbols.gmt subset was downloaded from the Molecular Signatures Database^41^ (http://www.gsea-msigdb.org/gsea/downloads.jsp) to evaluate related pathways and molecular mechanisms based on gene expression profiles and phenotypes. The minimum gene set function was set to 5 and the maximum gene set to 5,000 resamplings (5 × 1,000 resamplings). P < 0.05 and a false discovery rate (FDR) < 0.25 were considered statistically significant.

### Functional enrichment of DEGs

We used the GO annotation of genes in the ‘org.hs.eg.db’ v3.1.0 R software package as the background, then mapped genes into the background set, and used ‘clusterProfiler’ v3.14.3 for enrichment analysis to obtain the result of gene accumulation. The minimum gene set function was 5 and the maximum gene set was 5,000. *P* < 0.05 and an FDR < 0.25 were considered statistically significant.

### Bioinformatics validation of hub genes

The survival prediction of hub genes was assessed using Kaplan–Meier analysis with the ‘survival’ v3.2–7 R package. First, we obtained the DEG profile and prognostic data of 578 KIRC tumor samples from the TCGA and then determined the median expression value of each gene. Depending on whether the expression of a given gene was above or below the median, samples were assigned to either the "high expression" or "low expression" group. The log-rank test was used to evaluate the significance of the difference in survival between the high and low expression groups. If the test correlated with *P* < 0.05, we considered the gene to be a verified pivot gene.

Verifying that a gene is a DEG in most tumors is part of the broad-spectrum analysis. If gene is differentially expressed in most tumors, it means that it is broad-spectrum. Thereafter, a single gene is analyzed for its expression in tumors based on its differential expression in each tumor. We downloaded a unified standardized pan-cancer dataset from the UCSC (https://xenabrowser.net/) database: TCGA TARGET GTEx (PANCAN, *N* = 19,131, *G* = 60,499), and further extracted ENSG00000155066 (prominin-2; *PROM2*) and ENSG00000147872 (perilipin-2; *PLIN2*) gene expression data in each sample. We screened samples from the datasets for Solid Tissue Normal, Primary Solid Tumor, Primary Tumor, Normal Tissue, Primary Blood-Derived Cancer-Bone Marrow, and Primary Blood-Derived Cancer-Peripheral Blood. Thereafter, we performed log2(x + 0.001) transformation on each expression value. Lastly, we eliminated cancer types with less than 3 samples to obtain the expression data of 34 cancer types. We used R software (version 3.6.4) to calculate the expression difference between normal and tumor samples for each tumor and used the unpaired Wilcoxon Rank Sum and Signed Rank Tests to analyze the significance of the difference^36,41^.

### Statistical analysis

In this study, all statistical analyses were performed using R language (version 4.0.2). A two-sided P value of < 0.05 was considered as statistically significant. The adjusted P value was determined by the Benjamini–Hochberg method. All methods were carried out in accordance with relevant guidelines and regulations.

### Human and animal rights

This article only collected data from existing databases without any direct involvement of human participants.

## Results

### Data pre-processing

**Figure 2:**
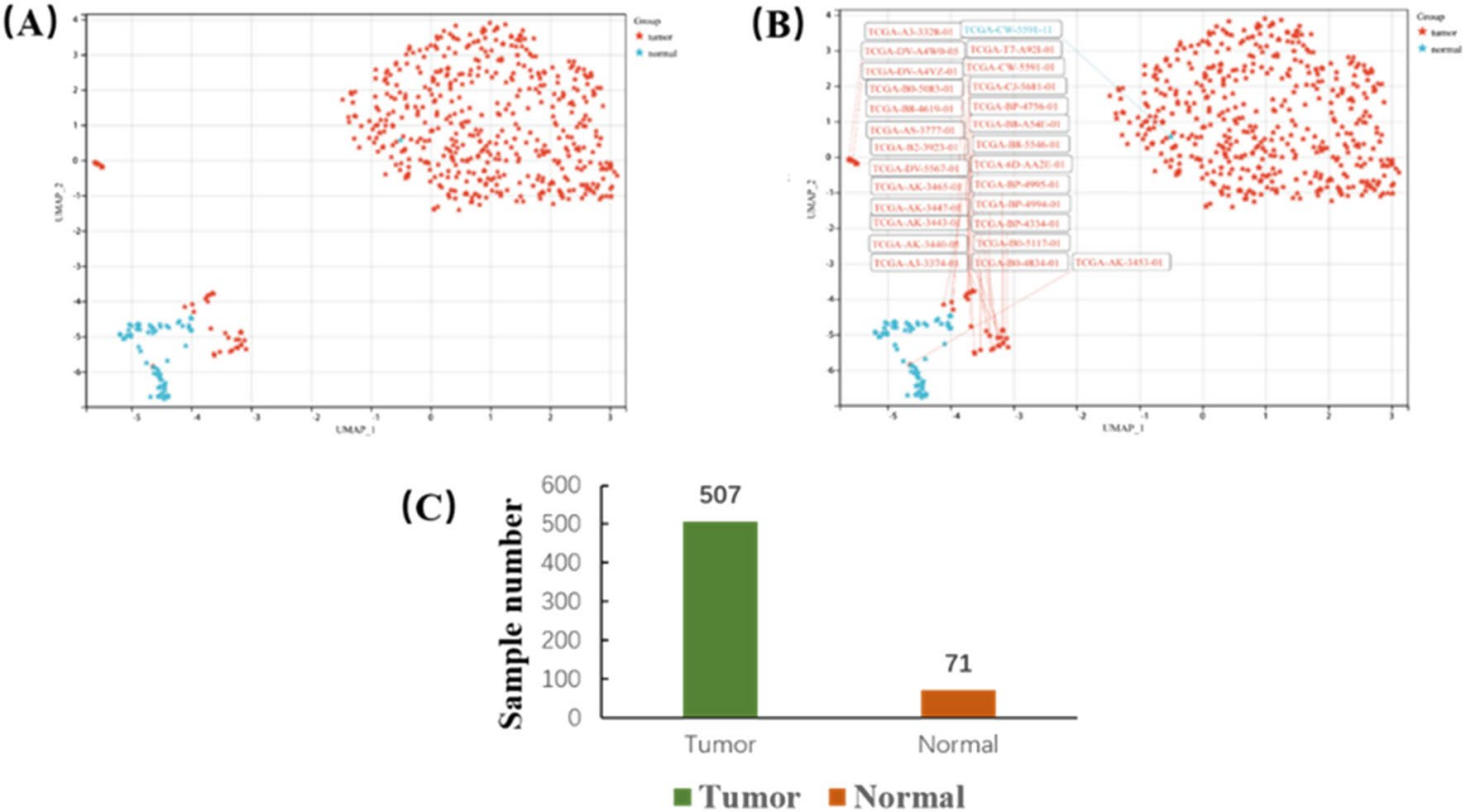
Identification of 71 normal and 507 KIRC samples by PCA. (**A**) Principal component analysis. Red dots represent tumor samples; blue dots represent normal samples. (**B**) Exclusion of 26 tumors and 1 normal sample after PCA. (**C**) Principal component analysis result.

The KIRC gene expression profile dataset downloaded from the TCGA database contains 72 normal samples and 534 tumor samples (Supplementary Table S1). Based on PCA, the first two principal components differentiated well between tumors and normal samples, forming two distinct clusters. In grouping tumor and normal samples, those with close distances had similar properties, and samples that deviated significantly from the population were removed. In total, 27 tumor tissues and 1 normal tissue were excluded (Fig. 2A,B). The gene expression profiles of the remaining 578 samples (Fig. 2C) and 259 ferroptosis-related genes were used for subsequent analysis.

### Identification of DEGs

**Figure 3:**
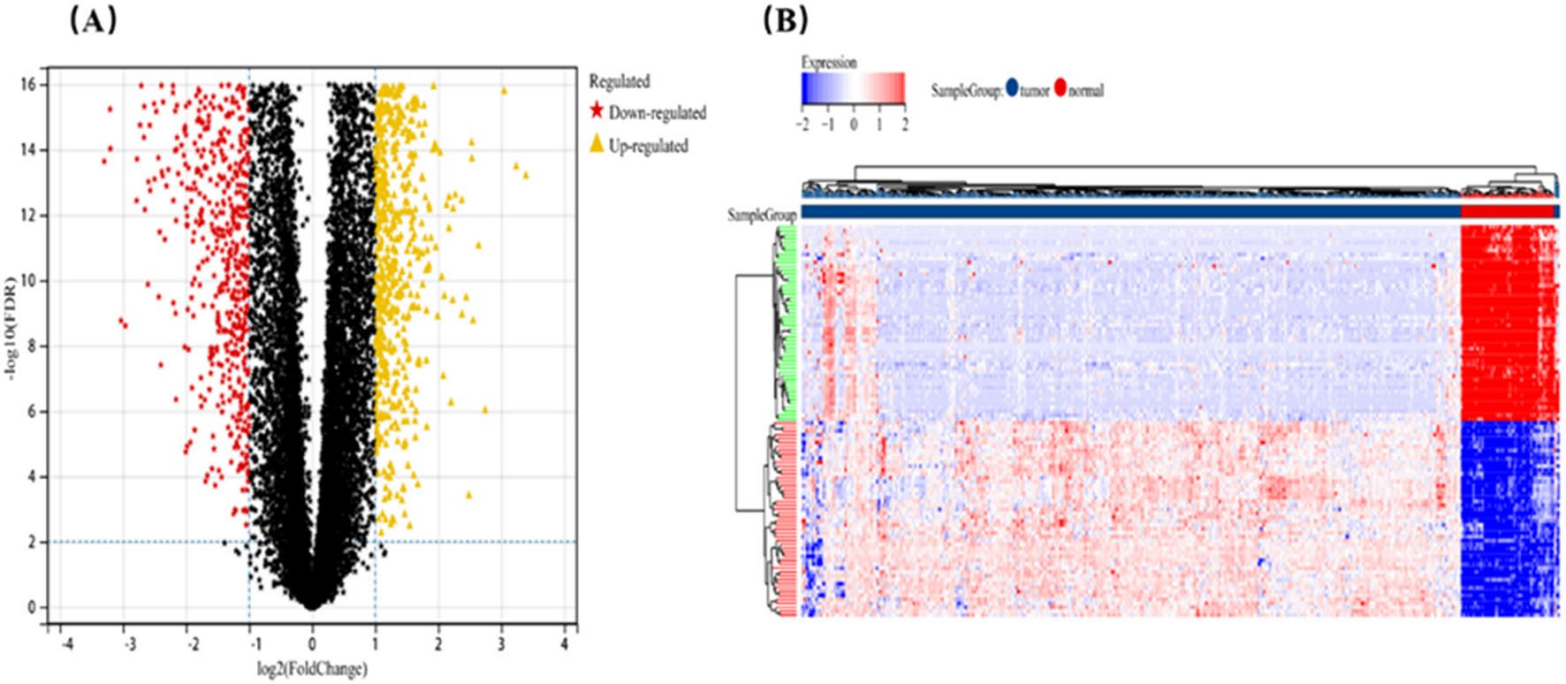
Identification of DEGs between 71 normal and 507 KIRC samples. (**A**) The volcano plot. Red dots represent down-regulated genes, black dots not-significant genes, and yellow dots up-regulated genes. (**B**) The heat map shows the expression difference of differentially expressed genes (DEGs).

The data set after excluding outliers was analyzed by limma analysis. From the results of the volcano plot and heatmap (Fig. 3A,B), we found 5,151 DEGs with obvious differences after analysis, of which 2,522 genes were up-regulated and 2,629 genes down-regulated.

### GSEA

**Figure 4:**
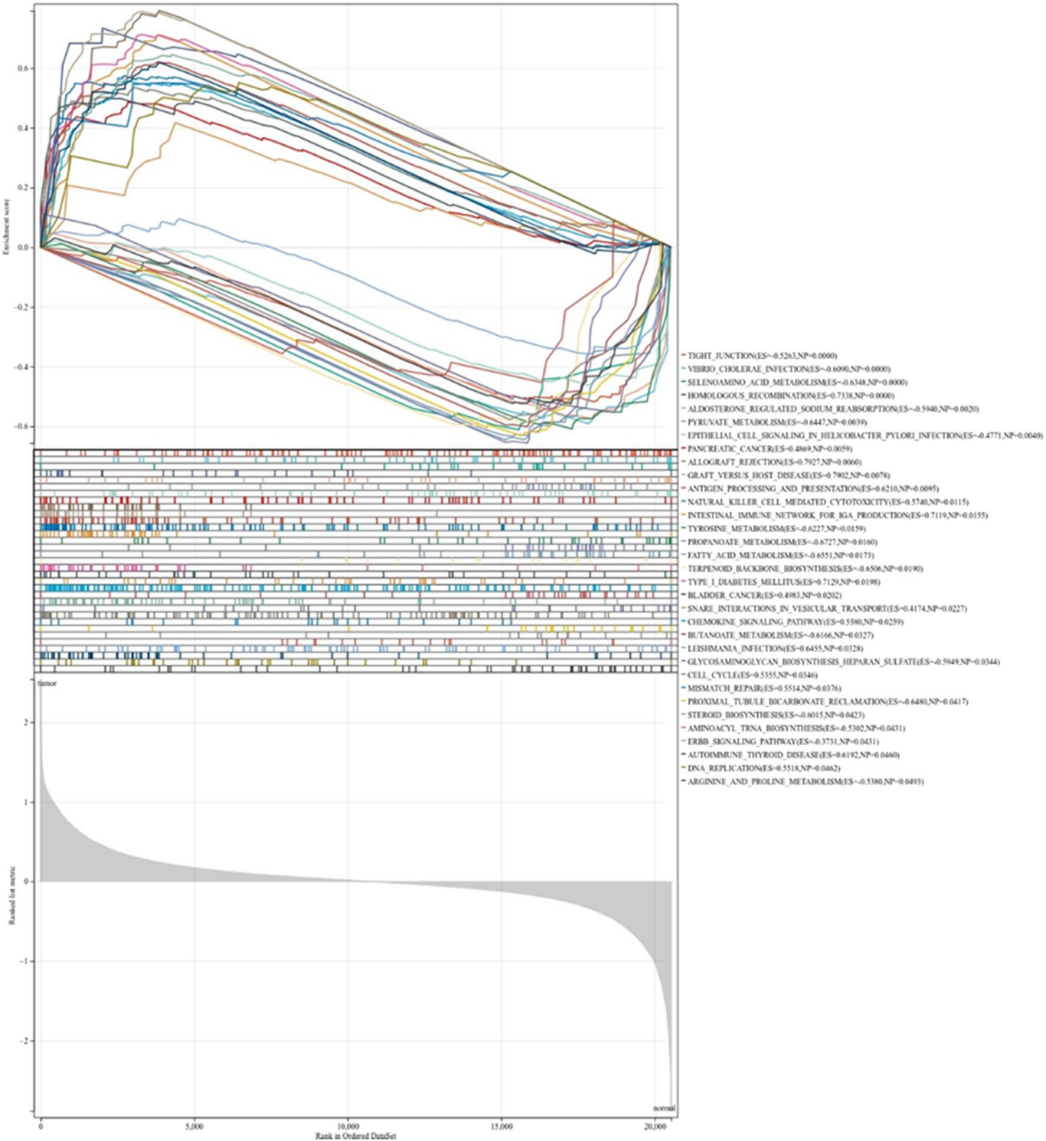
Gene set enrichment analysis.

GSEA is used to evaluate the distribution trend of genes in a predefined gene set ranked by phenotype correlation (based on its contribution to phenotype). As expected, several ferroptosis-related functions were involved, including pyruvate metabolism, propanoate metabolism, fatty acid metabolism, bladder cancer, butanoate metabolism proximal tubule bicarbonate reclamation steroid biosynthesis aminoacyl T-RNA biosynthesis, arginine and proline metabolism, etc. (Fig. 4) (www.kegg.jp/kegg/kegg1.html) ^42–44^.

### WGCNA and identification of critical modules

**Figure 5:**
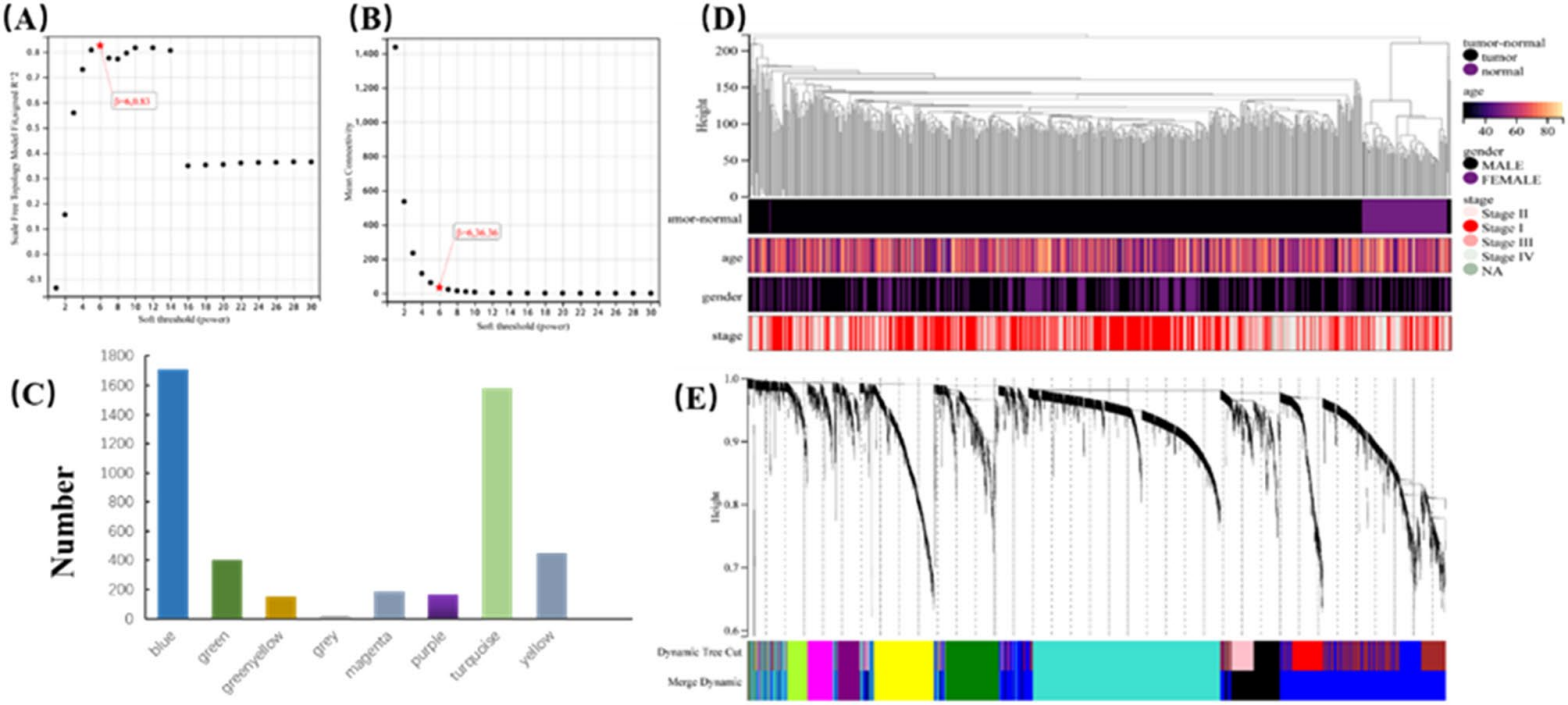
WGCNA of DEGs and identification of hub genes. (**A**) Scale independence. (**B**) Average connectivity. (**C**) Numbers of genes in the nine modules. (**D**) Clustering dendrogram of 578 samples. Black color represents ‘tumor’ for the variable ‘tumor-normal’; ‘Female’ or ‘Male’ for the variable ‘gender’; and ‘Stage I’, ‘Stage II’, ‘Stage III’, and ‘Stage IV’ for the variable ‘stage’. (**E**) Dendrogram. Each branch represents a gene, and each color represents a co-expression module.

**Figure 6:**
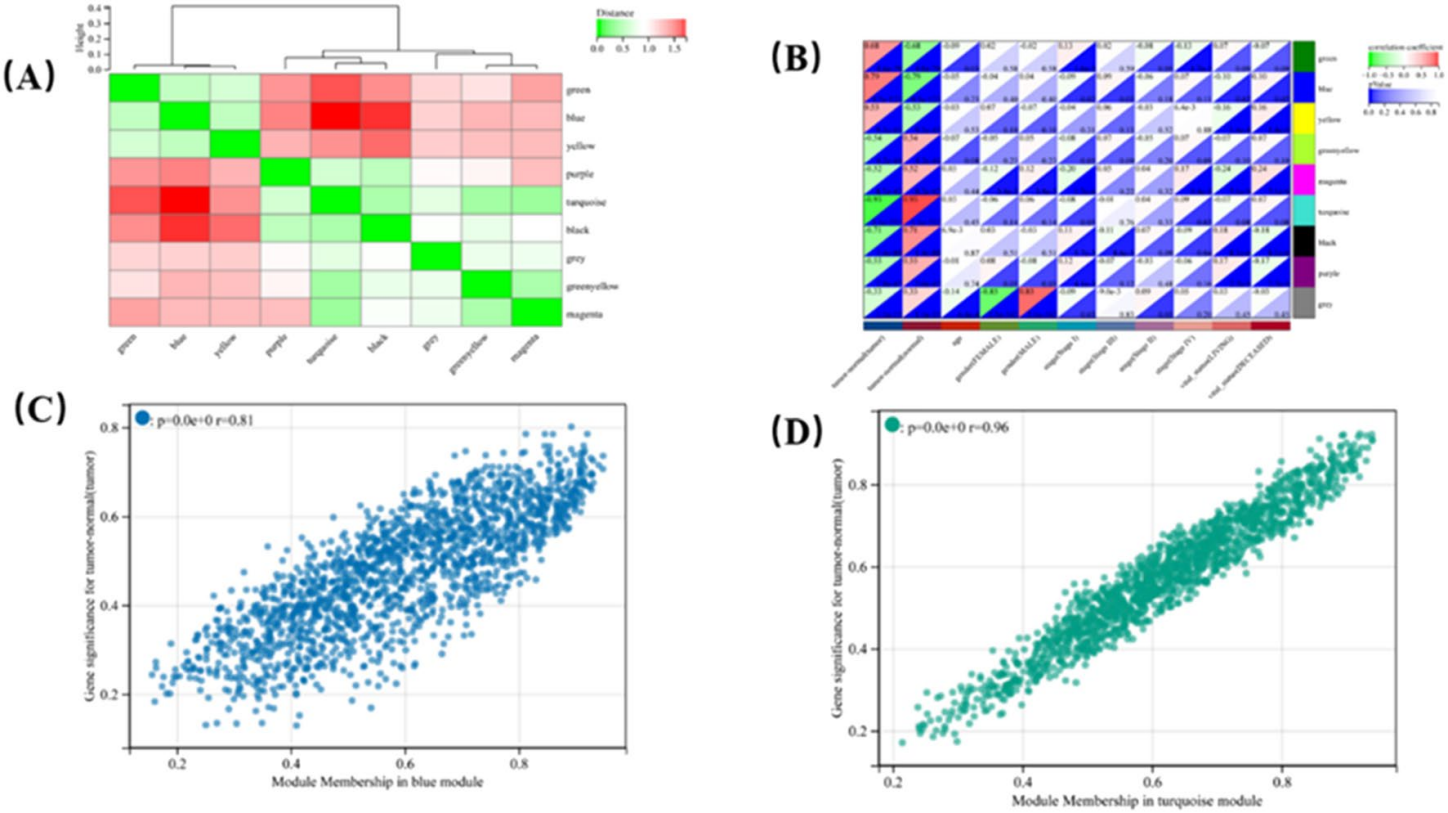
WGCNA of DEGs and identification of hub genes. (**A**) Module eigengene (MEs) heatmap. (**B**) Heatmap of the correlation between module eigengenes (MEs) and clinical characteristics of patients with KIRC. Each cell contains the correlation coefficient and *P*-value. (**C**, **D**) Scatter plots of GS score and MM (see the ‘Materials and methods’ section) for genes in the (**C**) blue and (**D**) turquoise modules.

We performed a cluster analysis according to the expression matrix of 5,151 DEGs and the network of clinical data of 578 KIRC samples. It can be seen that all samples in the cluster were within the cut-off threshold (height < 200) which means that all values were normal (Fig. 5D). Four clinical variables were used in WGCNA (Fig. 5D): tumor-normal, gender, age, and stage. The 578 samples were divided into two clusters: tumor and normal. To build a scale-free network, we set the soft threshold power β to 6, the independence to 0.83, and the average connectivity to 36.36 (Fig. 5A,B). The DEGs with similar expression patterns were gathered into the same module, and the modules showing the cutting height difference < 0.25 were merged. This process produced nine co-expression modules: blue, green, green-yellow, grey, magenta, purple, turquoise, black and yellow (Fig. 5C). The characteristic genes of the turquoise module (*r* = 0.96) and blue module(r = 0.81) are highly correlated with KIRC (Fig. 6C,D). These results were also confirmed by analysis of hierarchical clustering, heatmap, and adjacency relationship (Fig. 6A,B). Taken together, these results indicate that turquoise and blue modules may be closely related to KIRC. Therefore, the central genes of the turquoise and blue modules were further analyzed.

### Identifying candidate hub genes from the most relevant modules

As shown in Fig. 6C,D, the MM and GS scores were strongly correlated with each other in the turquoise and blue modules. The criteria for selecting pivot genes were relatively lower than the standard cut-off threshold (MM > 0.79). Under the unified threshold of "cor. gene Module Membership" > 0.6 and "cor. gene Trait Significance" > 0.6, a total of 708 genes were determined to be satisfied in the turquoise module and 357 genes in the blue module.

### Screening of co-expressed genes of ferroptosis-related genes and KIRC DEGs

**Figure 7:**
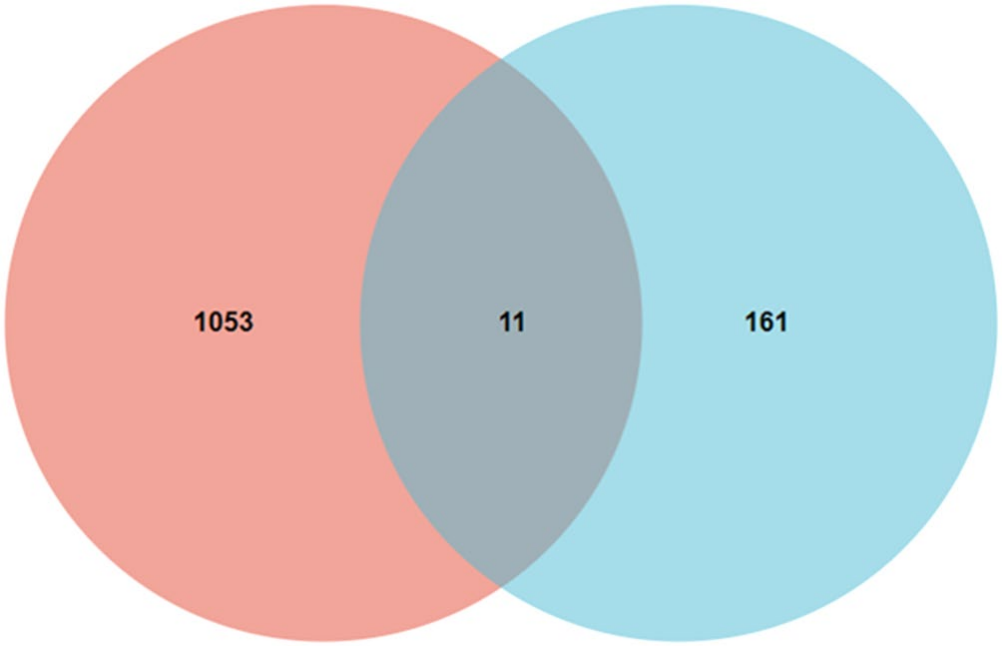
The Venn diagram demonstrating the relationship between ferroptosis genes and DEGs.

**Figure 8:**
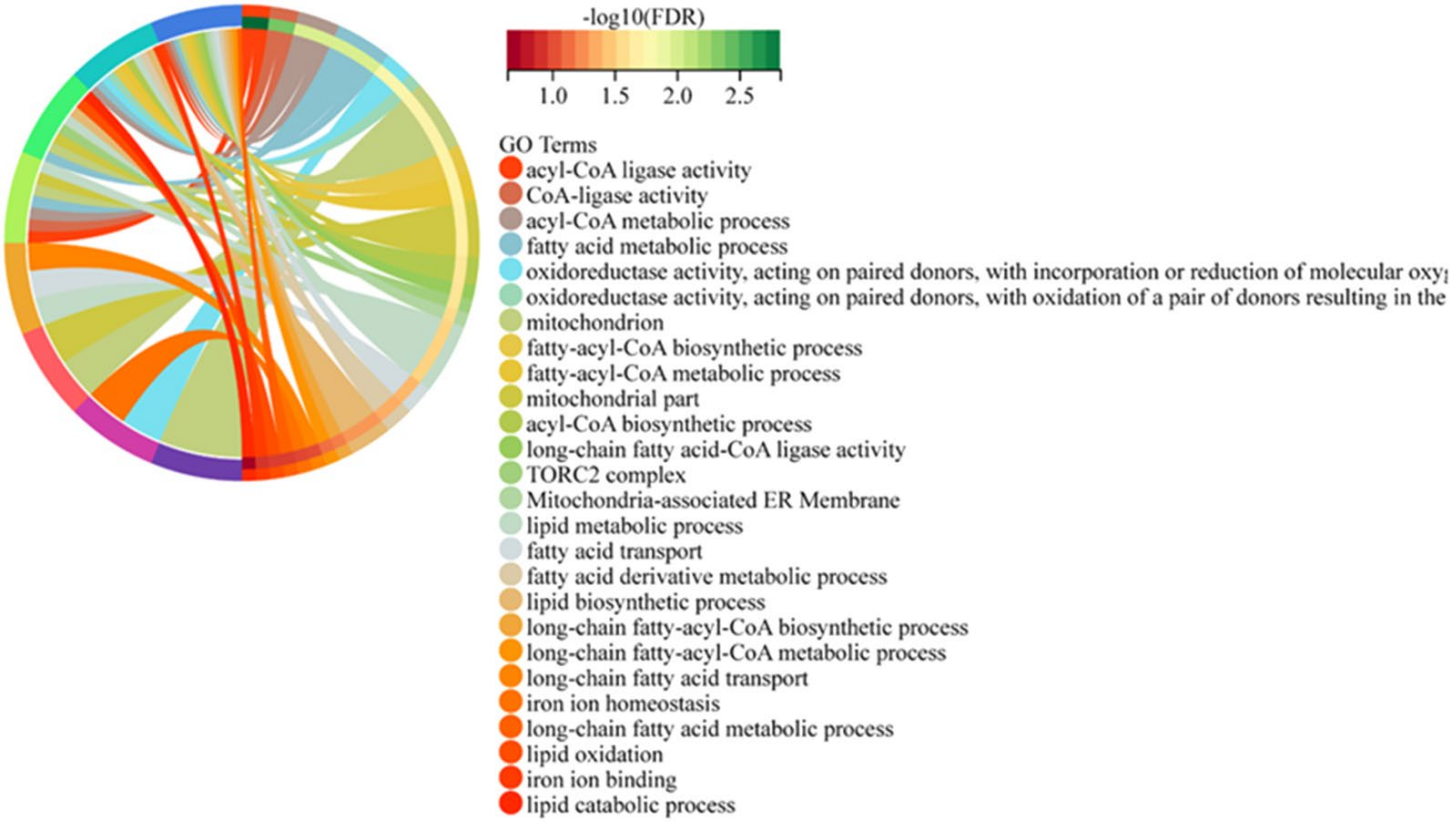
GO analysis of 11 co-expressed genes between ferroptosis-related genes and KIRC DEGs.

By analyzing the ferroptosis genes contained in the DEGs, we found that there were a total of 11 KIRC DEGs related to ferroptosis (Fig. 7). As shown in Fig. 8, the GO enrichment analysis of these 11 genes was all related to lipid metabolism. It is well known that abnormal lipid metabolism is an important factor inducing ferroptosis. In other words, ferroptosis plays a vital role in KIRC.

### Hub gene expression and correlation with survival

**Figure 9:**
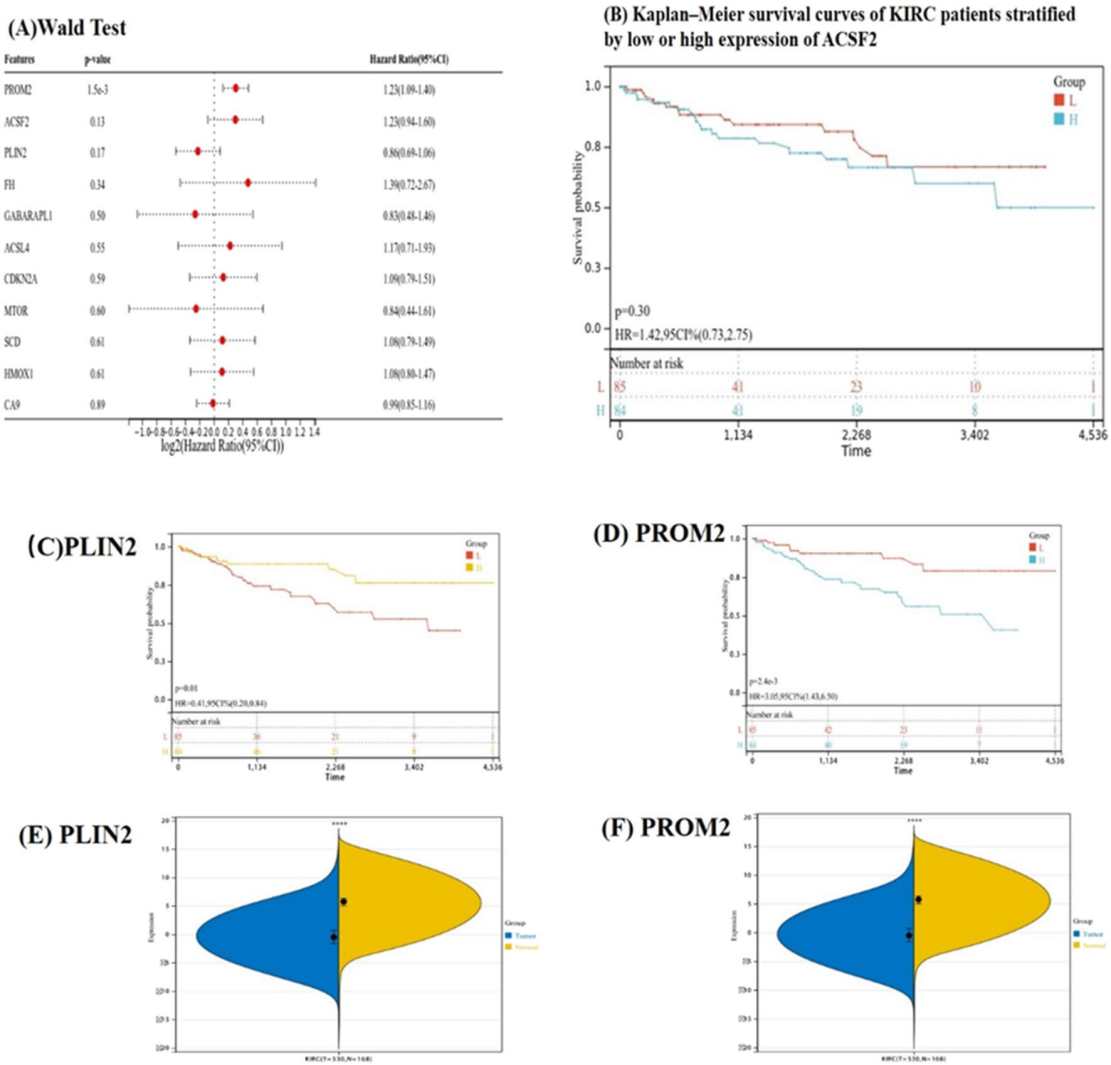
Survival analysis and validation of hub genes. (**A**) Wald Test. (**B**) Kaplan–Meier survival curves of patients with KIRC stratified by low or high expression of *PLIN2*. (**C**, **D**) Kaplan–Meier survival curves of patients with KIRC stratified by low or high expression of *PLIN2* (**C**) and *PROM2* (D). (E–F) Differences in expression of the *PLIN2* (**C**) and *PROM2* (**D**) genes between normal and tumor tissues. *P < 0.01.

The Wald test method was used to analyze the hub genes with *ACSF2*, *PROM2*, and *PLIN2* confirmed as possible prognostic-related genes (Fig. 9A). By examining the potential correlation between the expression of candidate central genes and patient survival, we found that *PROM2* and *PLIN2* were significantly related to prognosis (Fig. 9C,D), while no significance was found for *ACSF2* (Fig. 9B). Therefore, we defined the *PROM2* and *PLIN2* genes as the "final" pivotal genes.

We also confirmed that the expression of *PROM2* and *PLIN2* were significantly different between the normal and KIRC tissues (Fig. 9E,F). *PLIN2* was down-regulated in KIRC, while *PROM2* was up-regulated.

**Figure 10:**
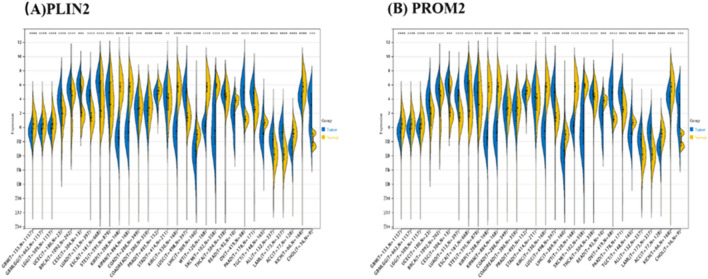
Validation of hub genes in various tumors. (**A**, **B**) Differences in expression of the *PLIN2* (**A**) and *PROM2* (**B**) genes in various tumors. *P < 0.01.

**Figure 11:**
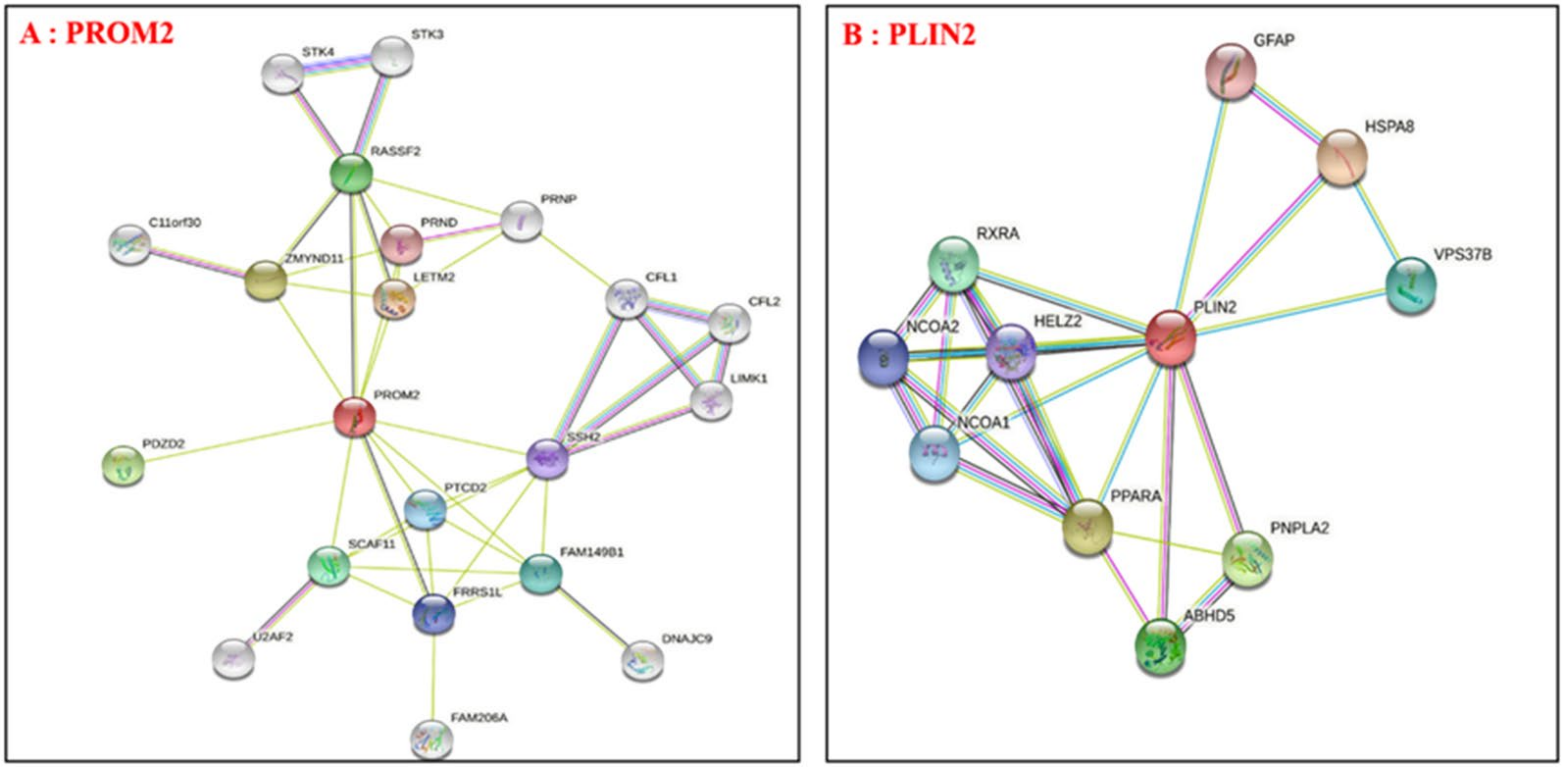
Protein–protein interaction of PROM2 (**A**) and PLIN2 (**B**).

As seen from Fig. 10A,B, the expression of *PROM2* and *PLIN2* were significantly different between multiple tumors. Furthermore, the protein–protein interaction network analysis chart is shown in Fig. 11A,B.

## Discussion

KIRC is one of the most serious malignant tumors worldwide. Various studies have used WGCNA to explore molecular markers for diagnosis and prognosis. Specifically, Zou et al. showed that KIRC has a unique metabolic state more prone to ferroptosis in response to the hypoxia-inducible factor pathway^45^. On the therapeutic front, they indicated GPX4 as an effective therapeutic target for KIRC. However, their research only focused on specific genes in KIRC, and the mechanism of ferroptosis in KIRC remained uncertain.

In this study, we found 5,151 DEGs between normal and KIRC tissues using limma analysis, indicating that the occurrence and development of KIRC are regulated by a complex genetic network. By using WGCNA, we identified that genes of the turquoise and blue modules were most closely related to KIRC, with 1,066 relevant genes. By analyzing these 1,066 KIRC-related genes and 259 ferroptosis genes, a total of 11 ferroptosis co-expression hub genes were found. Then, GO function enrichment analysis showed these genes were mainly involved in abnormal fatty acid metabolism pathways which regulate ferroptosis in KIRC—consistent with previous related studies. These findings on the importance of fatty acid metabolism pathways in ferroptosis may be helpful to understand the tumorigenic mechanism and treatment options of KIRC. Finally, our survival analysis of these 11 hub genes showed that *PROM2* and *PLIN2* expression are closely related to the poor prognosis of patients with KIRC. Therefore, *PROM2* and *PLIN2* may be promising prognostic indicators for patients with KIRC.

PLIN2, also known as adipose differentiation-related protein, wraps lipid droplets and phospholipids, as well as participates in the storage of neutral lipids in lipid droplets^46,47^. Ma et al. showed that the expression of *PLIN2* decreased significantly in the iron overload group, iron overload caused ferroptosis in the liver of mice with a decrease in GPX4 expression and an increase in Ptgs2 expression, resulting in a high level of lipid peroxidation, 79% decrease in the protein level of Perilipin-2 (PLIN2)^48^. Another study showed that PLIN2 promotes the proliferation and apoptosis of gastric cancer cells by modifying the ferroptosis pathway, such as regulating various ferroptosis-related genes, including acyl-CoA synthase long-chain family member 3, etc. *PLIN2* has also been shown to be an indispensable factors for inhibiting ferroptosis caused by abnormal fat metabolism in gastric cancer^49^. However, no research has been done on PLIN2 in KIRC. In line with previous studies, our findings confirm the critical role of *PLIN2* in ferroptosis inhibition caused by abnormal fat metabolism and further indicate *PLIN2* as a potential prognostic risk factor of KIRC.

Studies have shown that PROM2 participates in the composition of the plasma membrane microstructure where it mainly participates in biological processes such as promoting cell growth, migration, and perception by interacting with membrane cholesterol^50^. *PROM2* expression may be closely related to both prostate and breast cancer^51^. PROM2 is also a candidate gene marker for distinguishing renal chromophobe cell carcinoma and benign renal eosinophiloma^52^. Nevertheless, no research had been done on the role of PROM2 in KIRC before our study.

A variety of signaling pathways are involved in the regulation of tumor proliferation, migration, and invasion, including the JAK-STAT, NF-κB, Ras-Raf-MAPK, and Notch signaling pathways amongst others. Specifically, the phosphatidylinositol 3-kinase/protein kinase B (PI3K/AKT) signaling pathway is most commonly dysregulated in human tumors^53–57^, which plays an important role in a series of cell biological processes such as cell growth, proliferation, and angiogenesis^58–62^. In this regard, studies have shown that PROM2 can promote the proliferation, migration, and invasion of breast cancer by activating the PI3K/AKT pathway. Furthermore, ferroptosis plays an important role in triggering inflammation by activating the PI3K/AKT signaling pathway^63–67^. In our study, we found that PROM2 is an independent risk factor for KIRC and may have potential as a prognostic indicator. Moreover, we propose that PROM2 regulates ferroptosis via the PI3K/AKT signaling pathway in KIRK.

## Conclusion

In this study, we combined bioinformatics analysis and data set cross-validation to study the prognostic genes related to ferroptosis in KIRC and obtained two new hub genes (*PLIN2* and *PROM2*) that may be related to the prognosis of KIRC. We specifically elucidated the role of ferroptosis in KIRC, provided new insight into the molecular mechanism of KIRC, as well as revealed prognostic indicators and novel therapeutic targets for KIRC.

## Supplementary Information

Supplementary Information 1.

Supplementary Information 2.

Supplementary Information 3.

Supplementary Information 4.

## Abbreviations

DEGs: Differentially expressed genes
FDR: False discovery rate
GSEA: Gene set enrichment analysis
KIRC: Kidney renal clear cell carcinoma
OS: Overall survival
PCA: Principal component analysis
PI3K/AKT: Phosphatidylinositol 3-kinase/protein kinase B
PLIN2: Perilipin-2
PROM2: Prominin-2
RCC: Renal cell carcinoma
RNA-seq: RNA sequencing
TCGA: The Cancer Genome Atlas
TOM: Topological overlap matrix
WGCNA: Weighted gene co-expression network analysis
